# Secondary Prevention Using Cholesterol-Lowering Medications in Patients with Prior Atherosclerotic Cardiovascular Disease Events: A Retrospective Cohort Analysis

**DOI:** 10.36469/001c.28934

**Published:** 2022-01-19

**Authors:** Xue Han, Steven Fox, Michelle Chu, Jeff McCombs

**Affiliations:** 1 Department of Pharmaceutical and Health Economics, School of Pharmacy Leonard Schaeffer Center for Health Policy and Economics, University of Southern California; 2 Titus Family Department of Clinical Pharmacy, School of Pharmacy University of Southern California

**Keywords:** secondary prevention, atherosclerotic cardiovascular disease, cholesterol-lowering medications, hospitalization risk

## Abstract

**Background:** Secondary prevention with lipid-lowering medications in patients with atherosclerotic cardiovascular disease (ASCVD) is known to reduce the risk of clinical events and death. Current guidelines codify recommendations for implementing secondary prevention in appropriate patients. However, in real-world practice, secondary prevention is frequently initiated only after the patient experiences a cardiovascular-related hospitalization. The impact of these delays is not well known.

**Objectives:** To estimate the effects of delaying treatment on the risk of cardiovascular-related hospitalization and on costs for patients who meet the criteria for secondary prevention as specified in the 2013 American College of Cardiology/American Heart Association (ACC/AHA) Guidelines for Treatment of Blood Cholesterol to Reduce Atherosclerotic Cardiovascular Risk in Adults.

**Methods:** This is a retrospective cohort analysis using Humana data. Eligible patients were categorized by treatment group: (1) patients who initiated treatment before an ASCVD-related hospitalization and (2) patients who either did not initiate treatment until after an ASCVD hospitalization or never initiated treatment. The associations between the timely initiation of cholesterol-lowering medications for secondary prevention and (1) the risk for an ASCVD hospitalization and (2) health-care costs over one year, were estimated using multivariate regressions.

**Results:** A total of 272 899 secondary prevention patients were identified who met study selection criteria. Early treatment was associated with significant reductions in the risk of an ASCVD hospitalization at any time following the identification of the patient’s eligibility for secondary prevention (by 33% compared to those treated late or never, *P*<.0001), but was significantly associated with higher total cost over the first post-index year (by US $509, *P*<.001). Patients whose low-density lipoprotein cholesterol (LDL-C) levels were >130 mg/dL experienced higher ASCVD hospitalization risks, and also larger risk reductions if treated before an ASCVD hospitalization compared to patients with lower LDL-C levels who were treated late or never treated.

**Conclusions:** More widespread implementation of the treatment policies specified in the 2013 ACC/AHA Guidelines for secondary prevention should significantly reduce cardiovascular disease hospitalizations and reduce costs.

## INTRODUCTION

Cardiovascular disease (CVD) is the leading cause of death in the United States, resulting in significant health and economic burdens.^1^ The clinical research literature has documented (and medical opinion now accepts) that treatment with cholesterol-lowering medications significantly decreases cardiovascular risks and mortality when used for secondary prevention in patients who have already experienced a CVD-related event.^2–6^ The 2011 Adult Treatment Panel III Guidelines defined secondary prevention patients as having elevated low-density lipoprotein cholesterol (LDL-C) levels and a previous coronary heart disease event.^7^ This “treat earlier” policy was updated in 2013 by the American College of Cardiology/American Heart Association (ACC/AHA), based on growing research literature documenting the efficacy of cholesterol-lowering drug therapy for secondary prevention.^8^ However, several studies have reported that this policy, promoting the expansion of secondary prevention for CVD, remains poorly implemented in clinical practice.^5,9,10^ Piepoli et al and Cheng et al found that only a small proportion of heart attack survivors, and patients after revascularization surgery, initiated secondary prevention.^11,12^ Conversely, Bellows et al and Yao et al found that commercial plan patients with atherosclerotic cardiovascular disease (ASCVD) did appear more adherent to statin therapy after the release of new guidelines.^13,14^ Also, recent research reinforces that pretreatment LDL levels impact the cardiovascular risk reduction benefit that statin treatment confers.^15,16^

The purpose of this study is to document the potential harm done by the suboptimal implementation of the secondary prevention treatment policies, as detailed in the 2013 ACC/AHA Guidelines. This analysis estimates the impact of delayed initiation of secondary prevention, including delaying treatment for the newly defined (ie, expanded) high-risk population specified in the 2013 ACC/AHA Guidelines. The study replicates methods used in our earlier analysis of delayed primary prevention.^17^ In this analysis, we use pre-2013 paid claims data to identify patients who met the 2013 ACC/AHA Guidelines criteria for secondary prevention based on their observed treatment history. We estimate the impact of “on time” initiation of secondary prevention on the risks of both ASCVD and non-ASCVD hospitalizations, and on health care costs, relative to patients who either delayed treatment or never initiated drug therapy. While pre-2013 data maximizes the number of observable “untreated” primary prevention patients, this analysis does not estimate the extent to which the 2013 ACC/AHA Guidelines are currently implemented, nor how implementation rates have evolved over time.

## METHODS

### Data Sources

This was a retrospective cohort analysis using data derived from the Humana claims database, covering January 1, 2007, to June 30, 2013. The Humana claims database is a national and longitudinal database covering 22 million persons in the United States and includes Humana’s Commercial and Medicare Advantage enrollees and contains medical (inpatient, outpatient, and emergency room), pharmacy, and laboratory data (including test results).^18^ Using these data enables identification of patients who meet the new criteria for secondary prevention, but who may not have been treated, during the period prior to publication of the 2013 ACC/AHA Guidelines. Data on these patients allows estimation of the impact of treating patients meeting the criteria for secondary prevention, before they experience an ASCVD hospitalization (on-time treatment) and estimation of the cost of delaying or foregoing treatment. These data do not, however, support estimating the current rate of compliance to the 2013 ACC/AHA Guidelines.

Data elements include administrative claims from medical encounters (inpatient, outpatient, ambulatory, emergency room), pharmacy dispensing, demographics, enrollment eligibility, and laboratory test values (for a subsample of members). These data can be used to derive relatively complete diagnostic and drug utilization profiles for each patient that reflect the patient’s health status at the point in time when each patient becomes eligible for secondary prevention.

### Study Cohort

Patients included in the analytic sample met the requirements for secondary prevention, as specified in the 2013 ACC/AHA Guidelines, based on a chronological assessment of their diagnostic and procedure history. Specifically, secondary prevention patients were identified once the patient was found to have ≥1 medical claim with a diagnosis of ASCVD or had a paid claim for a CPT-4/ICD-9-DM procedure service directly related to an ASCVD diagnosis.^19^ The complete list of included diagnoses and ASCVD-related procedures is provided in the **Online Supplemental Material, Table S1**. ASCVD diagnoses included: acute myocardial infarction (AMI), unstable angina, subarachnoid and intracerebral hemorrhage, stroke, transient cerebral ischemia, cerebrovascular disease, atherosclerosis, peripheral vascular disease, arterial embolism, and arterial disorder.^10^ ASCVD-related procedures included: coronary artery bypass graft surgery (CABG), coronary angioplasty, revascularization surgery, and peripheral bypass. The earliest date on which the patient was assessed as having qualified for secondary prevention using the 2013 ACC/AHA Guidelines criteria was assigned as that patient’s index date. In general, the index date was defined by the occurrence of a qualifying ASCVD event. It is possible that patients with qualifying diagnoses or procedures that occurred either prior to obtaining Humana coverage or before the beginning of the available data period (2007) would be missed.

### Inclusion and Exclusion Criteria

Once identified, study patients were screened to determine if there was a minimum of 6 months of continuous enrollment before their index date, with both medical and pharmacy coverage, and that their enrollment continued for 12+ months following their index date. This latter selection criterion allowed for the evaluation of first-year costs but also required that patients survived at least one year post-identification. These requirements limited the index dates included in the study to the period from July 1, 2007, to June 30, 2012. These data restrictions allowed the analysis to develop variables correlated with the baseline health status and follow-up outcomes for study patients. Patients who filled or refilled a prescription for any treatment for high cholesterol before their index date were excluded, as were patients younger than 21 years old on their index date.

All-cause health care costs were measured for each patient over each patient’s first post-index year. The post-index ASCVD events were quantified by counting days to both the first ASCVD-related and non-ASCVD hospitalization, not restricted to the one-year post-index period. Those time-to-event outcomes were evaluated using Cox proportional hazards models.

### Key Explanatory Variables and Other Covariates

Patients were categorized as having received timely secondary prevention if they initiated cholesterol-lowering medication at any point following their index ASCVD event, but before experiencing any subsequent ASCVD-related hospitalization. Timely secondary prevention was entered as a dichotomous variable in the analysis. The comparison group consisted of individuals who did not fill any cholesterol-lowering prescriptions or who filled their initial post-index prescription only after experiencing an ASCVD-related hospitalization. All available cholesterol-lowering treatments were used to define secondary prevention status (statins, bile acid sequestrants, cholesterol absorption inhibitors, fibric acid derivatives, niacin, or omega-3 fatty acid ethyl esters). Since PCSK9 inhibitors received FDA approval after the end of the study data period, they were not included.

The covariates used to adjust baseline risk in the statistical models included: age, gender, race, region, benefit group, health plan type (Medicare, Medicaid, health maintenence organization, point of service, preferred provider organization), diagnostic and prescription histories, prior hospital admissions, and baseline all-cause health care costs (health insurance payments plus out-of-pocket costs) as measured over the 6 months prior to the patient’s index date. The specific ASCVD diagnosis or event that triggered the index date was entered as a covariate in the analysis; a patient could have more than one type of “index” ASCVD diagnosis on the same index date. Baseline LDL-C levels were also collected for the subgroup of patients with available lab result data. The LDL-C record closest to the index date was selected as the patient’s LDL-C level at baseline.

### Outcomes

This analysis investigated three primary outcomes: (1) an ASCVD-related hospitalization and time from index date to this ASCVD hospitalization; (2) a non-ASCVD-related hospitalization and time from index date to this non-ASCVD hospitalization; and (3) all-cause health care costs measured over the first year following the index date. Follow-up data was censored either when continuous enrollment of both pharmacy and medical insurance ran out, or an outcome-defining event occurred. ASCVD-related hospital admissions were identified using the ICD9 and CPT codes specified in the **Online Supplemental Material, Table S1**. Inpatient services were used to define outcome ASCVD events in order to more precisely distinguish new events from routine follow-up outpatient services related to the original index event that qualified the patient for secondary prevention. All-cause health care costs were measured as the combination of insurance payments and the patient’s out-of-pocket charges over the first year following the patient’s index date and were sub-classified by type of service (medical, pharmacy, and total).

### Statistical Analysis

Descriptive statistics comparing patients’ baseline characteristics across treatment status used either the Pearson’s chi-square test or Student’s t-test to identify significant differences as appropriate.^20,21^ The impact of secondary prevention on the risk of cardiovascular-related and non-CVD hospitalization was estimated using Cox proportional hazards models, which allow the analysis of all post-index events up until the patient’s eligibility expired, including death.^22^ The assumed appropriateness of a Cox model was validated using a Kaplan-Meier curve indicating the hazard ratio (HR) of the two groups is proportional over time. The impact of secondary prevention on the costs for medical, pharmacy and total health care services over the first year were estimated using generalized linear models (GLM) with gamma distribution and log link.^23^ All models adjusted for age, gender, race, region, index ASCVD event, health plan type, diagnostic history, prescription drug use history, prior hospital admission, and health service costs.

Two sub-analyses were also performed to test the results from the Cox proportional hazard models. The first sub-analysis re-estimated the effect of timely secondary prevention on CVD and non-CVD hospitalizations using the subset of patients with baseline LDL data. This sensitivity analysis only included patients who had LDL levels at baseline and was used to test the association between treatment effect and baseline LDL-C. The LDL-C lab results collected closest to index date (either pre-index date or within 5 days following index date) was assigned as patients’ baseline LDL. The LDL levels were classified into four categories consistent with the 2011 Adult Treatment Panel Guidelines: LDL-C <101 mg/dL, 101-130 mg/dL, 131-160 mg/dL, and >160 mg/dL.

The second sub-analysis further investigated possible differential treatment effects of on-time secondary prevention treatment variable and the patients’ baseline LDL levels. All analyses were performed using the SAS System for Unix, Version 9.3.

## RESULTS

**Figure 1.**
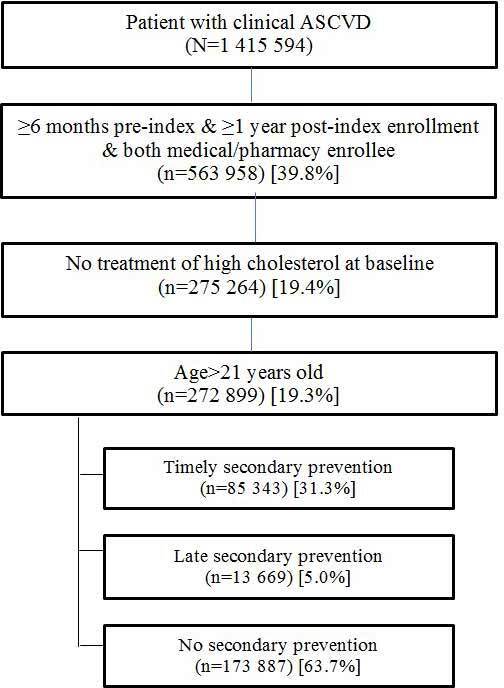
Funnel Chart of Patient Selection, Based on Inclusion/Exclusion Criteria Abbreviation: ASCVD, atherosclerotic cardiovascular disease.

A flow diagram outlining patient selection for the study is presented in Figure 1. Just over 1.4 million patients were identified with ASCVD, of whom 593 958 met the selection criteria for 18 months of data surrounding their index date. The final sample comprised 272 899 patients over age 21 who were eligible for secondary prevention, based on criteria specified in the 2013 ACC/AHA Guidelines. Nearly 64% of patients identified as qualifying for secondary prevention (prior to the ACC/AHA Guideline’s publication in 2013) never filled any prescriptions for a cholesterol-lowering medication, and an additional 5% filled a prescription only after a second (ie, post-index) CVD-related hospitalization. Stated differently: complete implementation of the treatment policies in the 2013 ACC/AHA Guidelines would have more than tripled the number of patients receiving secondary prevention compared with the pre-Guideline period analyzed here.

Unadjusted comparisons of baseline characteristics between patients who did and did not receive timely secondary prevention are shown in Table 1. The average duration of follow-up data was 1078 days (2.95 years) for patients with timely secondary prevention, versus 803 days (2.2 years) for those with delayed or no secondary prevention. Patients who initiated timely secondary prevention treatment were mostly male, age 55-74, and qualified for secondary prevention due to experiencing an AMI, unstable angina or having undergone a CVD-related surgical procedure. Patients treated “on time” also differed from patients with delayed therapy or no therapy on most other characteristics shown in Table 1. Somewhat surprisingly, patients who initiated timely secondary prevention displayed lower unadjusted rates of all-cause hospitalization, and lower all-cause health costs over the 6-month period prior to becoming eligible for secondary prevention (ie, they would appear to have been at lower risk).

**Table 1.** Descriptive Demographics by Treatment Status: Treated Before CVD-related Hospitalization, vs Late/Never Treated

**Characteristics**	**Timely Secondary Prevention (n=85 343) [31.3%]**	**Late/No Secondary Prevention (n=187 556) [68.7%]**	***P* Value**
**Age at year of index date, %**			
22-44 years	2.4%	5.5%	<0.0001
45-54 years	7.9%	7.9%	
55-64 years	14.8%	12.0%	
65-74 years	45.3%	37.8%	
>75 years	29.6%	36.8%	
**Sex, %**			
Men	51.3%	46.2%	<0.0001
**Index diagnosis, %**			
AMI	21.1%	11.9%	<0.0001
Unstable angina	11.4%	5.9%	<0.0001
Hemorrhage	1.3%	2.1%	<0.0001
Stroke	28.8%	27.6%	<0.0001
Transient cerebral ischemia	10.6%	10.4%	0.0558
Cerebrovascular disease	11.7%	16.4%	<0.0001
Atherosclerosis	7.4%	8.7%	<0.0001
Peripheral vascular disease	25.6%	32.0%	<0.0001
Arterial embolism	0.5%	0.4%	0.0438
Arterial disorder	0.9%	0.9%	0.3029
CABG	3.2%	0.5%	<0.0001
Coronary angioplasty	9.8%	2.0%	<0.0001
Revascularization surgery	0.03%	0.01%	<0.0001
Peripheral bypass	0.16%	0.17%	0.3844
**Race, %**			
White	70.0%	68.9%	<0.0001
Black	10.4%	9.2%	
Asian	0.5%	0.4%	
Hispanic	1.8%	1.3%	
Unknown	17.4%	20.2%	
**Region, %**			
Midwest	23.5%	29.1%	<0.0001
Northeast	1.8%	2.3%	
South	64.5%	59.2%	
West	9.1%	8.7%	
Unknown	1.2%	0.7%	
**Health plan, %**			
Medicare	85.8%	83.5%	<0.0001
Medicaid	0.4%	0.4%	
HMO	3.8%	4.0%	
POS	2.8%	3.1%	
PPO	5.7%	5.6%	
Other plan	1.6%	3.4%	
**Prior utilization (6 months)**			
Hospital admission, %	10.1%	12.9%	<0.0001
Medical costs, US $, mean (SD)	1864 (4253)	2257 (5491)	<0.0001
Inpatient costs, US $, mean (SD)	1408 (8084)	2170 (12 457)	<0.0001
Pharmacy costs, US $, mean (SD)	667 (1477)	651 (1898)	0.0326
Total costs, US $, mean (SD)	3939 (10 202)	5079 (14 754)	<0.0001
**LDL values at baseline in subgroup (n=49 224)**			
**Newly eligible secondary prevention patients**	**n=14 381**	**n=34 843**	
<101 mg/dL	30.6%	45.3%	<0.0001
101-130 mg/dL	34.5%	35.3%	
**ATP III eligible secondary prevention patients**			
131-160 mg/dL	23.8%	14.8%	
>160 mg/dL	11.1%	4.6%	

Abbreviations: AMI, acute myocardial infarction; ATP, Adult Treatment Panel; CABG, coronary artery bypass grafting surgery; CVD, cardiovascular disease; HMO, health maintenance organization; LDL, low-density lipoprotein; POS, point of service; PPO, preferred provider organization.

For the subgroup of patients who had baseline LDL data available, early prevention patients exhibited higher LDL-C levels, relative to both untreated patients and patients who delayed secondary prevention. This is consistent with the ACC/AHA Guidelines prior to 2013, which recommended treatment of cholesterol-lowering medications mainly just for patients with elevated LDL-C levels.

Over 88% of patients who initiated treatment at any time in the post-index period used statins as their cholesterol-lowering medication of choice. Approximately 68% of statin patients were specifically prescribed moderate intensity treatment. In addition, 2.9% of treated patients used a combination of a statin and non-statin as their regimen for secondary prevention. (See **Online Supplemental Material, Table S2)**.

Table 2 presents unadjusted data for the study outcomes, which must be viewed with caution given the need to adjust for the significant baseline risk differences in the two study populations. The unadjusted rate of CVD-related hospitalizations was lower for patients initiating timely secondary prevention therapy, both in the first post-index year (6.8% vs 9.6%) and when measured using the patient’s complete post-index data (16.7% vs 17.0%). Conversely, patients initiating early treatment had nearly equal rates of non-CVD hospitalizations during the first year (22.7% vs 22.5%), but higher non-CVD hospitalization rates (versus patients with delayed or no treatment) when measured using all of the patient’s available post-index data (43.5% vs 40%). However, patients initiating timely secondary prevention also had higher medical costs, inpatient costs, and pharmacy costs over the first year following index date.

**Table 2.** Unadjusted Patient Outcomes, by Treatment Status

**All Risk Groups**	**Timely Secondary Prevention (n=85 343) [31.3%]**	**No Secondary Prevention (n=187 556) [68.7%]**	***P* Value**
**Proportions of events, %**			
CVD-related hospitalization	16.7	17.0	<0.0001
Non-CVD hospitalization	43.5	40.0	<0.0001
CVD hospitalization in 1st year	6.8	9.6	<0.0001
Non-CVD hospitalization in 1st year	22.7	22.5	0.3507
**Time to events in days, median (SD)**			
Time to CVD-related hospitalization	962 (574)	698 (515)	<0.0001
Time to non-CVD hospitalization	698 (586)	575 (507)	<0.0001
Time to end of enrollment	1078 (549)	803 (506)	<0.0001
**Health care cost over 1st year post in US $, mean** (**SD**)			
Medical	7177 (11 933)	7019 (13 211)	<0.0001
Inpatient	12 071 (27 612)	9474 (28 898)	<0.0001
Pharmacy	2262 (3706)	1631 (4566)	<0.0001
Total	21 511 (33 386)	18 124 (35 645)	<0.0001

Abbreviations: CVD, cardiovascular disease; SD, standard deviation.

The results of all multivariate analyses of the effects of early treatment are summarized in Table 3, for ease of comparison. Absolute risk reductions are also reported in Table 3 and were calculated using the number of patients with that outcome, divided by the number of people in each appropriate group. The primary Cox model results for hospitalization indicated that early initiation of secondary prevention was associated with an adjusted relative risk reduction of 33% in the risks of ASCVD-related hospitalization (HR=0.67 [CI 0.66-0.69]). This reduction in relative risk translates into a reduction in the absolute risk of experiencing a CVD hospitalization of 0.26%. Also, the results indicated that initiating timely secondary prevention was not associated with any altered risk of a non-ASCVD hospitalization (HR=1.00 [CI 0.98-1.01]).

**Table 3.** Summary of Estimated Effects of Secondary Prevention

			**Clinical Outcomes**			
	**CVD-Related Hospitalization**			**Non-CVD-Related Hospitalization**		
	**HR**		**95% CI**	**HR**	**95% CI**	
**Baseline Cox model of hospitalization risk (n=272 899**)						
Timely secondary prevention	0.67^a^		[0.66-0.69)	1.00	(0.98-1.01)	
**Adding baseline LDL levels for subpopulation (n=49 224**)						
Timely secondary prevention	0.68^a^		**(**0.64-0.72)	1.05^a^	(1.01-1.08)	
101-130 mg/dL (vs <101 mg/dL)	1.02		**(**0.96-1.08)	0.91^a^	(0.88-0.94)	
131-160 mg/dL (vs <101 mg/dL)	1.16^a^		**(**1.08-1.25)	0.87^a^	(0.83-0.92)	
>160 mg/dL(vs <101)	1.45^a^		**(**1.31-1.60)	0.95	(0.88-1.02)	
**Treatment effect (relative risk) in each LDL-C level group for subpopulation [n=49 224]**						
LDL <101 mg/dL	0.66^a^		**(**0.60-0.72)	1.04	(0.98-1.10)	
Change in absolute risk	**-0.55%**					
LDL 101-130 mg/dL	0.74^a^		**(**0.67-0.82)	1.12^a^	(1.06-1.19)	
Change in absolute risk	**-0.92%**					
LDL 131-160 mg/dL	0.65^a^		**(**0.57-0.73)	0.97	(0.89-1.05)	
Change in absolute risk	**-1.14%**					
LDL >160 mg/dL	0.61^a^		(0.51-0.73)	0.94	(0.83-1.07)	
Change in absolute risk	**-3.21%**					
**All-cause cost outcomes 1 year post**						
	**Medical+Inpatient Costs**		**Drug Costs**		**Total Costs**	
	**Effect in US $**	**95% CI**	**Effect in US $**	**95% CI**	**Effect in US $**	**95% CI**
**GLM model (n=272 899)**						
Secondary prevention	147	(-63 356)	272^a^	(246 297)	509a	(293 726)

Abbreviations: CVD, cardiovascular disease; GLM, generalized linear model; HR, hazard ratio; LDL, low-density lipoprotein; LDL-C, low-density lipoprotein cholesterol.

^a^ The parameters are statistically significant at a significance level of 0.05.

This estimate of the impact of timely secondary prevention on the risk of ASCVD-related hospitalization did not change significantly when the Cox model was re-estimated using only those patients with an available baseline LDL level (n=49 224), entering that LDL level into the Cox model as a covariate. Note that the relative risk of an ASCVD-related hospitalization increases monotonically with the patient’s baseline LDL level, whereas the risk of non–ASCVD-related hospitalizations was significantly higher (ie, averaged overall higher in patients with LDL data available, versus not available), driven mostly by a higher risk only in the lowest LDL group.

The absolute risk of an ASCVD-related hospitalization likely varies by LDL level, which implies that the effectiveness of primary prevention in reducing CVD-related hospitalization risk may also vary by baseline LDL. To investigate this possibility, the third analysis reported in Table 3 calculated the effect of timely secondary prevention in each LDL risk group using interaction terms between early treatment and LDL classification. These results found that the treatment effect, measured as a change in relative risk, is relatively constant across baseline LDL levels (estimated HR range: 0.61 to 0.74). However, absolute CVD hospitalization risk increases monotonically with the patient’s baseline LDL level (see previous model results in Table 3), which means that the reductions in absolute risk increase monotonically with baseline LDL, ranging from 0.55% in patients with LDL <101 mg/dL to 3.21% in patients with LDL >160 mg/dL.

The impact of secondary prevention on health care costs over the first post-index year was estimated using GLM (lower panel, Table 3). Secondary prevention was significantly associated with a higher total cost over one year of US $509 [95% CI: US $293-US $726] primarily due to increased prescription drug costs per patient of US $272 (95% CI: US $246-US $297). These estimates control for baseline cost differences, which were significantly lower for the timely secondary prevention group (see Table 1).

**Figure 2.**
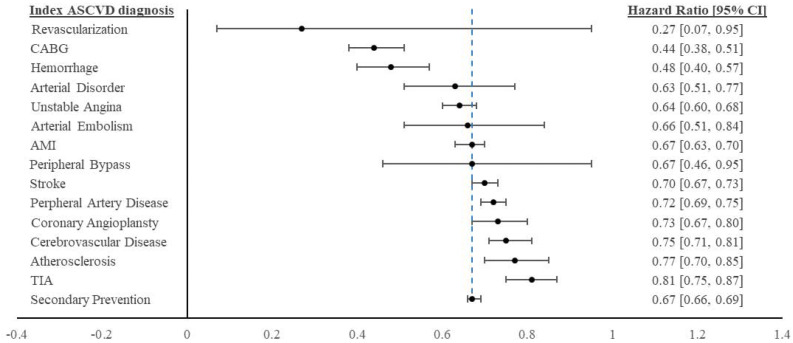
Summary Adjusted Effects of Timely Secondary Prevention Estimates Across Index ASCVD Risk Groups Abbreviations: AMI, acute myocardial infarction; ASCVD, atherosclerotic cardiovascular disease; CABG, coronary artery bypass grafting surgery; TIA, transient ischemic attack. The blue vertical line represents the general secondary prevention treatment effect for all ASCVD risk groups.

Finally, we re-estimated the impact of secondary prevention on CVD hospitalization risk for all patients, depending on their specific index ASCVD event or diagnosis. The treatment effects of secondary prevention vary across different ASCVD risk groups and are displayed graphically in Figure 2. The overall treatment effect reported in Table 3 is indicated at the bottom of the figure [HR=0.67]. These results indicated that timely secondary prevention may have had a more significant protective effect on the likelihood of a future CVD-related hospitalization among certain ASCVD risk groups. In particular, timely secondary prevention treatment was associated with larger reductions in CVD hospitalization if patients had experienced unstable angina, hemorrhage, arterial disorder, CABG, or revascularization in the pre-index period.

## DISCUSSION

The establishment and updating of treatment guidelines are common policy tools, aimed at improving patient outcomes and possibly lowering health care costs as health care technology and medical evidence changes over time. The impact of new guidelines on achieving those goals depends on three factors: the magnitude of individual benefits (on outcomes and costs) from guideline compliance; the size of the population affected by the guidelines (or guideline changes); and the completeness of their implementation. This study addresses the first two factors as they relate to the 2013 ACC/AHA Guidelines for Treatment of Blood Cholesterol to Reduce Atherosclerotic Cardiovascular Risk in Adults.^8^ Specifically, the criteria for identifying patients for secondary prevention detailed in the 2013 ACC/AHA Guidelines dropped the earlier requirement that secondary prevention patients exhibit an LDL-C level >130 mg/dL (with drug treatment optional for 100-129 mg/dL).

Using paid claims data for ASCVD patients from a data period before the publication of the 2013 ACC/AHA Guidelines, this study estimates that the new Guidelines increased the size of the population recommended for secondary prevention by nearly 69%, based on the data for the subgroup of patients with available lab results (see Table 1). Our results also estimate that 94% of these only later-eligible secondary prevention patients never initiated cholesterol lowering drug therapy while the remaining 6% delayed treatment until after experiencing a new ASCVD-related hospitalization. This study also found that the timely initiation of drug therapy, consistent with 2013 ACC/AHA Guidelines, had a significant and consistent impact—reducing hospitalization risk across all secondary prevention patients (HR range=0.61 to 0.74, Table 3). The timely initiation of drug therapy reduced the absolute risk of a CVD-related hospitalization by 0.55% for patients with a baseline LDL level less than 100 mg/dL. This benefit increased monotonically to a reduction in absolute risk of 3.21% for patients with a baseline LDL >160 mg/dL. Taken together, the results in Table 3 suggest that widespread implementation of the 2013 ACC/AHA Guidelines for secondary prevention could significantly improve overall patient outcomes.

However, this study also found cholesterol-lowering medication was associated with a higher annual cost of US $500 in the first post-index year, primarily due to increased prescription drug costs per patient. That result may not persist later, assuming that risk reductions accrue beyond the first year. Also, some of the lipid-lowering drugs remained on-patent during the study period. Generic versions are now available for many of these medications, which would reduce the estimated pharmacy cost differences reported here.

### Limitations

The decision to initiate timely secondary prevention may have been correlated with important but unmeasured risk factors that preferentially assigned patients into these two treatment groups. The first line of defense against this threat to study result validity is to expand the analysis to include all important patient baseline characteristics. This study documented the existence of many significant differences in the observable characteristics of treated and untreated patients. While our statistical analyses used this extensive set of characteristics as covariates, it remains impossible to fully control for treatment selection using any non-randomized approach. However, it is encouraging that after adjusting for all available risk factors, the risk of non–ASCVD-related hospitalizations differed only minimally between the timely and late/no prevention groups.

The number of patients receiving early or delayed secondary prevention may also be underestimated due to any missing statin prescription claims that were paid for using cash, rather than billed to insurance, given the relatively low cost of these medications. The implication is that some patients in the “no secondary prevention” group may have initiated cholesterol-lowering medications. The effect of that potential data gap would be to underestimate the effectiveness of treatment in reducing CVD-related outcomes, making the effect sizes presented here falsely too low.

Sensitivity analyses that included different subgroups of patients were performed to confirm the robustness of the main results, and to more clearly understand both the relative risks of CVD hospitalizations and the associated treatment effects across each LDL-C level subgroup. Since lab results reporting LDL-C levels were not available in the data for many patients, those analyses were only conducted on the smaller subpopulation with available baseline LDL-C results. The overall secondary prevention effect on CVD-related hospitalization risk did not change in this subgroup analysis, at least partially validating the results from the whole-sample analysis.

Patient adherence to cholesterol-lowering medications was also not directly considered in this analysis, only initial prescription fills. Again, to the extent that our early treatment population includes some nonadherent patients, this would tend to underestimate the effects of adherent early treatment. Future analyses should account for patients’ long-term adherence to medications, estimating the impact of duration of therapy rather than just the act of initiating treatment.^24^

## CONCLUSIONS

Implementation of the 2013 ACC/AHA Guidelines is intended to expand secondary prevention treatment to all ASCVD patients with a prior CVD event, regardless of their LDL-C levels. In this study using pre-Guideline data, we found that this policy change could benefit patients significantly, if Guideline policy recommendations are implemented fully in the newly eligible population. While timely secondary treatment benefits all patients, timely treatment was found here to benefit high-risk patients the most, at least in terms of absolute risk reduction. Patients with higher LDL levels exhibit the highest absolute risk of experiencing CVD-related hospitalization, and also experienced the largest absolute risk reductions after initiating cholesterol-lowering medications. The extent to which these Guidelines have been implemented since 2013 was not assessed here. This study provides numerical estimates documenting the magnitude of benefits gained, per patient, from more fully implementing secondary prevention.

### Conflict of Interest

There are no disclaimers.

## Supplementary Material

Online Supplemental Materials

Online Supplemental Materials
