# Supplementary material for: Secondary Prevention Using Cholesterol-Lowering Medications in Patients with Prior Atherosclerotic Cardiovascular Disease Events: A Retrospective Cohort Analysis
Source: J Health Econ Outcomes Res. 2022 Jan 19;9(1):11–9. doi: 10.36469/001c.28934 (PMC8770090; doi:10.36469/001c.28934)
Supplement: Online Supplemental Materials [file jheor_2022_9_1_28934_80488.pdf]

Journal of Health Economics and Outcomes Research

Online Supplementary Material

Secondary Prevention Using Cholesterol-Lowering Medications in Patients With Prior Atherosclerotic Cardiovascular Disease Events: A Retrospective Cohort Analysis. *JHEOR*. 2022;9(1):11-19. doi:10.36469/jheor.2022.28934

Table S1. List of ICD-9-CM Codes Used

Table S2. Patient-Level Use in Cholesterol-Lowering Medications

This supplementary material has been provided by the authors to give readers additional information about their work.

This is an open-access article distributed under the terms of the Creative Commons Attribution 4.0 International License (CCBY-4.0). View this license's legal deed at http://creativecommons.org/licenses/by/4.0 and legal code at http://creativecommons.org/licenses/by/4.0/legalcode for more information.

Table S1. List of ICD-9-CM Codes Used

Description	Code Type	Code
AMI	ICD-9-CM diagnosis	410.xx, 412.xx
Unstable angina	ICD-9-CM diagnosis	411.xx
Hemorrhage	ICD-9-CM diagnosis	430.x, 431.x, 432.x
Stroke	ICD-9-CM diagnosis	433.xx, 434.xx, 436.xx
Transient cerebral ischemia	ICD-9-CM diagnosis	435.xx
Cerebrovascular disease	ICD-9-CM diagnosis	437.xx,438.xx, 439.xx
Atherosclerosis	ICD-9-CM diagnosis	440.xx
Peripheral vascular disease	ICD-9-CM diagnosis	443.xx, 440.2
Arterial embolism	ICD-9-CM diagnosis	444.xx, 445.xx, 449.xx
Arterial disorder	ICD-9-CM diagnosis	446.xx, 447.xx, 448.xx
CABG	ICD-9-CM procedure	36.10, 36.11, 36.12, 36.13, 36.14, 36.15, 36.16, 36.19
	CPT codes	335.10-335.19, 335.21-335.23, 335.33-335.36, S2005-S2009
Coronary angioplasty	ICD-9-CM procedure	00.61, 00.62, 00.64, 00.65, 00.66, 00.40-00.48, 36.04, 36.06, 36.07, 36.09, 38.22
	CPT codes	372.05, 372.06, 372.36, 372.37, 372.46, 372.47, 354.52, 354.72, 354.76, 929.20-929.29, 929.33-929.38, 929.44, 929.73, 929.82, 929.84, 929.95, 929.96
Peripheral bypass	ICD-9-CM procedure	39.2x
	CPT codes	00.63
Revascularization procedure	ICD-9-CM procedure	36.2x, 36.3x, 36.9x

Table S2. Patient-Level Use in Cholesterol-Lowering Medications

	Total No. of Treated Patients (n=85 343)	
	**No.**	**%**
**Cholesterol-lowering agents (Rx=86 526)**		
Any statin	77 315	89.35%
Low-intensity	13 918	18.00%
Moderate-intensity	52 420	67.80%
High-intensity	10 977	14.20%
Bile acid–binding resins	2540	2.94%
Fibrates	3781	4.37%
Ezetimibe	2286	2.64%
Niacin	1332	1.54%
Omega-3 fatty acid	737	0.85%
**Treatment regimens (n=85 343)**		
Statin	74 779	87.62%
Non-statin	8066	9.45%
Combinations (statin and non-statin)	2498	2.93%
